# Facile Synthesis of Ag-Doped Urchin-like MnO_2_ on Carbon Cloth for Supercapacitors

**DOI:** 10.3390/ma17061312

**Published:** 2024-03-12

**Authors:** Yanqiu Feng, Henghui Qu, Yanxiang Wang, Lanzhong Wang, Yongbo Wang, Deli Yang, Bohan Ding, Yue Sun, Jinghe Guo, Shichao Dai

**Affiliations:** 1 Key Laboratory for Liquid-Solid Structural Evolution and Processing of Materials (Ministry of Education), Shandong University, Jinan 250061, China; 2 Shandong Hi-Speed Materials Technology Development Co., Ltd., Jinan 250014, China; 3 School of Foreign Languages and Literature, Shandong University, Jinan 250100, China

**Keywords:** α-MnO_2_, silver doped, composite electrode, transition metal oxides, electrochemical capacitor, structural property

## Abstract

Based on MnO_2_/carbon cloth (CC) composite materials, an Ag-doped MnO_2_ nanowire, self-assembled, urchin-like structure was synthesized in situ on the surface of CC using a simple method, and a novel and efficient flexible electrode material for supercapacitors was developed. The morphology, structure, elemental distribution, and pore distribution of the material were analyzed using SEM, TEM, XRD, XPS, and BET. The electrochemical performance was tested using cyclic voltammetry (CV) and galvanostatic charge/discharge (GCD). In the three-electrode system, GCD testing showed that the specific capacitance of the material reached 520.8 F/g at 0.5 A/g. After 2000 cycles at a current density of 1 A/g, the capacitance retention rate was 90.6%, demonstrating its enormous potential in the application of supercapacitor electrode materials.

## 1. Introduction

Under the dual pressure of fossil energy depletion and the limited development of new energy [1], developing new environmentally friendly energy and efficient storage devices, improving energy utilization efficiency, and reducing energy loss have become urgent tasks [2]. In recent years, researchers have conducted more and more research on batteries, but batteries undergo phase transitions during charging and discharging, resulting in relatively short cycle life and inability to meet the requirements of rapid charging and discharging [3]. Owing to the limited development of batteries, supercapacitors with longer lifespans and more environmentally friendly properties have emerged with the efforts of researchers worldwide. Supercapacitors have the advantages of short charging and discharging times, excellent cycling and rate performance, and can meet the needs of human life and production [4]. Therefore, they are considered the most competitive new energy storage devices of the next generation and have great development potential. Supercapacitors are composed of electrodes, electrolytes, and separators [5]. Electrode materials are a key component of supercapacitors and play a crucial role in their excellent performance [6]. According to the different discharge mechanisms of supercapacitors, supercapacitors can be divided into two categories: electric double-layer capacitor (EDLC) and pseudocapacitor (PC) [7]. The commonly used electrode materials for EDLC include carbon materials, while those for PC include transition metal compounds and conductive polymer materials. EDLC materials have a stable structure and excellent cyclic stability, but their specific capacitance is generally smaller. PC materials generally have high specific capacitance and energy density, but during the charging and discharging process, redox reactions occur, resulting in longer charging and discharging times, leading to poor cyclic stability [8,9].

Many high-performance supercapacitors have been developed using transition metal compounds with nanostructures [10], such as aluminum oxide [11], niobium oxide [12], iron oxide [13], etc. As a typical representative of pseudocapacitive materials, MnO_2_ has attracted great attention as a good candidate for electrode materials owing to its rich sources, low price, and excellent electrochemical performance (theoretical specific capacitance of 1370 F/g) [14,15]. MnO_2_ can obtain various forms under different process conditions, and its most common crystal forms are α-MnO_2_, β-MnO_2_, γ-MnO_2_, δ-MnO_2_, and λ-MnO_2_ [16,17,18]. These MnO_2_ isomers fully utilize their respective advantages based on their tunnel size and crystal type [19]. However, due to its low conductivity [20,21], there is a significant difference between the actual specific capacitance of MnO_2_ and the theoretical specific capacitance, which limits its further application. Therefore, developing some efficient and stable MnO_2_ electrode materials remains a major challenge. In order to address the shortcomings of MnO_2_-based electrode materials, extensive efforts have been made to improve their electrochemical performance. The research work can be roughly divided into two parts. The first part is devoted to the preparation of nano MnO_2_, which improves the ion diffusion rate by increasing the specific surface area. The second part is to composite MnO_2_ with conductive materials such as carbon fibers and carbon nanotubes, graphene, conductive polymers, etc. [22,23,24,25], in order to solve the shortcomings of a single component. Murat Cakici [26] used a green hydrothermal method to coat coral-like MnO_2_ nanostructures on carbon fiber fabric, fully utilizing the advantages of MnO_2_ coral structure and carbon fiber fabric and improving the pseudocapacitive performance of the material. The specific capacitance of the composite material can reach 467 F/g at 1 A/g. The supercapacitor prepared using CFF/MnO_2_ exhibited excellent energy density of 20 Wh/kg at a power density of 175 W/kg and also exhibited excellent electrochemical performance. Haribandhu Chaudhuri [27] prepared a novel bi-phasic MnO_2_ nanoflower structure of reduced graphene oxide composite containing α-MnO_2_ nanorods and δ-MnO_2_ nanosheets. The specific capacitance of rGO@α-MnO_2_/rGO@δ-MnO_2_ reaches 267 F/g at 1 A/g. After 10,000 cycles, the capacitance retention rate is 83%, demonstrating good specific capacitance and excellent cyclic ability. Although compounding with these conductive materials improved the electrochemical performance of the material, it did not significantly improve the intrinsic conductivity of MnO_2_. Doping metals (Co, Fe, Na, Zn, Ni, Cu, Al) in MnO_2_ can enhance the stability of crystals [28,29,30,31,32]. The higher hole concentration in these crystals expands the specific surface area, resulting in faster diffusion of electrons and ions during the redox reaction [33]. Without any interface limitations, the intrinsic conductivity of MnO_2_ is improved by changing physical interactions and crystal structure, thereby increasing capacitance. Saheed A. Adewinbi [34] used a binder-less electrode preparation method to deposit Co-doped MnO_2_ and Cu-doped MnO_2_ on ITO glass substrate. Co@MnO_2_ film electrode exhibits remarkable specific capacitance, indicating that the incorporation of Co into MnO_2_ electrode material can enhance charge transfer and transport. Thus, the capacitive activity of MnO_2_ electrode material is relatively improved. Among various conductive materials, Ag has excellent conductivity and surface activity and is a good choice as a doping element. For example, Yang Liu [35] compounded Ag with MnO_2_ as a dopant using the sol-gel method and deposited it on the ITO glass substrate to form a composite film. When the Ag doping concentration is 7.5%, the specific capacitance is 306 F/g at 1 A/g. B. Mazinani [36] synthesized MnCo_2_O_4_ (MCO) and Ag-doped MCO spinel oxide nanoparticles using the combustion method. Therefore, preparing Ag-doped MnO_2_ using a simple and economical method remains a huge challenge. In addition, reducing production costs and using environmentally friendly methods to prepare electrode materials are still research hotspots.

In this work, urchin-like spheres formed by Ag@MnO_2_-nanowire self-assembly on the surface of carbon cloth (CC) were synthesized using a simple method at room temperature (CRTMOA). Catalysis, doping, and in situ composite were directly completed during the preparation process at room temperature. The effects of Ag^+^ concentration on the morphology and coverage of Ag@MnO_2_ nanowires grown on CC were investigated. The results show that when the Ag^+^ concentration is 3%, the specific capacitance of CRTMOA is 520.8 F/g at 0.5 A/g, which is higher than that of RTMOA (urchin-like spheres of Ag@MnO_2_ synthesized at room temperature without growth on CC). Multiple charge and discharge tests on CRTMOA3 show that its capacitance retention rate is 90.6% after 2000 cycles. All these indicate that the prepared Ag@MnO_2_/CC self-supporting flexible electrode has great potential in the application of supercapacitor electrodes.

## 2. Experiment

### 2.1. Materials

Manganese sulfate (MnSO_4_) and ammonium persulfate ((NH_4_)_2_S_2_O_8_) were purchased from China National Pharmaceutical Group Chemical Reagent Co., Ltd. (Shanghai, China), ammonium sulfate ((NH_4_)_2_SO_4_) and silver nitrate (AgNO_3_) were purchased from Aladdin reagent (Shanghai, China), and polyvinylpyrrolidone (PVP) was purchased from Tianjin Kemio Chemical Reagent Co., Ltd. (Tianjin, China). All reagents were analytically pure and used directly without purification.

### 2.2. Preparation of Ag-Doped MnO_2_ at Room Temperature (RTMOA)

First, 1.2 g MnSO_4_, 1.83 g (NH_4_)_2_S_2_O_8_, 1.98 g (NH_4_)_2_SO_4_, and 0.05 g PVP were added to 40 mL of deionized water and stirred evenly. The molar ratios of 13.6 mg, 40.8 mg, and 68 mg AgNO_3_ to the final synthesis of MnO_2_ were 0.01, 0.03, and 0.05, and the reaction was conducted at 25 °C for 24 h. After the reaction was completed, it was washed three times with deionized water and ethanol and filtered through suction, and then the precipitate was vacuum dried at 60 °C to obtain the products, which were recorded as RTMOA1, RTMOA3, and RTMOA5, respectively.

### 2.3. Preparation of Ag@MnO_2_/CC Composite Materials at Room Temperature (CRTMOA)

**Figure 1:**
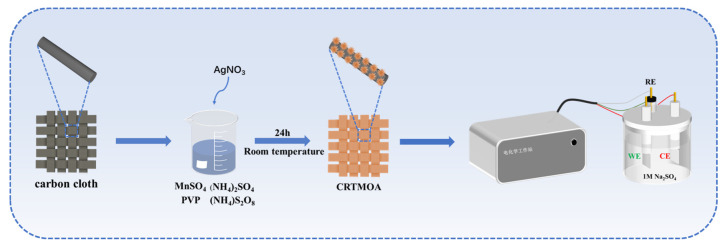
Schematic of the fabrication process of CRTMOA and the electrochemical measurement process.

The entire preparation and electrochemical measurement process is shown in Figure 1. Firstly, CC was cut into 2 × 2 cm pieces, washed ultrasonically with deionized water and ethanol for 15 min, and then dried in a vacuum oven at 60 °C. Next, 1.2 g MnSO_4_, 1.83 g (NH_4_)_2_S_2_O_8_, 1.98 g (NH_4_)_2_SO_4_, and 0.05 g PVP were added to three beakers containing 40 mL deionized water and stirred evenly. Then, 13.6 mg, 40.8 mg, and 68 mg AgNO_3_ were added successively, so that the molar ratios of AgNO_3_ to the final synthesis of MnO_2_ were 0.01, 0.03, and 0.05. The above-mentioned clean CC was then added, and the reaction was conducted at 25 °C for 24 h. After the reaction was completed, it was washed three times with deionized water and ethanol and filtered through suction, and then the CC attached to the sediment was vacuum dried at 60 °C to obtain the products, which were recorded as CRTMOA1, CRTMOA3, and CRTMOA5, respectively. Due to the low concentration of Ag^+^, the catalytic effect was relatively poor, and the growth of MnO_2_ on the CC was not ideal, resulting in there being almost no difference between the quality of CRTMOA1 and the quality of the CC before the reaction. The masses of MnO_2_ grown on CRTMOA3 and CRTMOA5 were 1.4 mg and 4 mg, respectively.

During the above preparation process, it was found that the addition of Ag^+^ not only increased the conductivity of the material but also rapidly catalyzed the reaction. This is because Ag^+^ can serve as an efficient catalyst for accelerating the oxidation of metal ions in aqueous solutions containing S_2_O_8_^2−^ [37].

### 2.4. Characterization

Field emission scanning electron microscopy (FESEM, SU-70, Hitachi, Tokyo, Japan) was used to observe the surface morphology of the material, and energy dispersive spectrometry (EDS, Hitachi, Tokyo, Japan) was used to analyze the types and contents of elements in the micro-region. The microstructure of the prepared samples was observed using transmission electron microscopy (TEM, JEM-2100, JEOL, Tokyo, Japan). The phase structure of the samples was characterized using an X-ray diffractometer (XRD, DMAX-2500PC, Rigaku, Tokyo, Japan) at diffraction angles of 10° to 80°. X-ray photoelectron spectroscopy (XPS, EscaLab 250Xi, Thermo Fisher Scientific, Waltham, MA, America) was used to analyze the types of elements and bonding modes on the surface of materials. Fourier transform infrared spectroscopy (FTIR, Vector 33, Billerica, MA, USA) was used to characterize the functional group types of the samples, and a specific surface area tester (BET, Tristar II 3020, micromeritics, Norcross, GA, USA) was used to analyze the specific surface area and pore size distribution of the materials.

### 2.5. Electrochemical Measurements

The electrochemical testing was completed using an electrochemical workstation (CHI660E, Chenhua, Shanghai, China), and the electrochemical performance measurements were conducted using cyclic voltammetry (CV), galvanostatic charge/discharge (GCD), and cyclic stability at room temperature. The test was conducted using a three-electrode system, using the prepared materials as the working electrode (WE), a platinum electrode as the counter electrode (CE), a saturated calomel electrode as the reference electrode (RE), and 1 mol/L Na_2_SO_4_ solution as the electrolyte. According to the GCD curve, the specific capacitance of the electrode under different current densities was calculated according to the equation $C_{s}=I∆t/m∆V$, where $C_{s}$ (F/g) represents the mass specific capacitance, I (A) represents the voltage-dependent current, ∆t (s) represents the discharge time, m (g) represents the mass of the active material per electrode sheet, and ∆V (V) represents the voltage interval.

## 3. Results and Discussion

### 3.1. Surface Morphology Analysis

**Figure 2:**
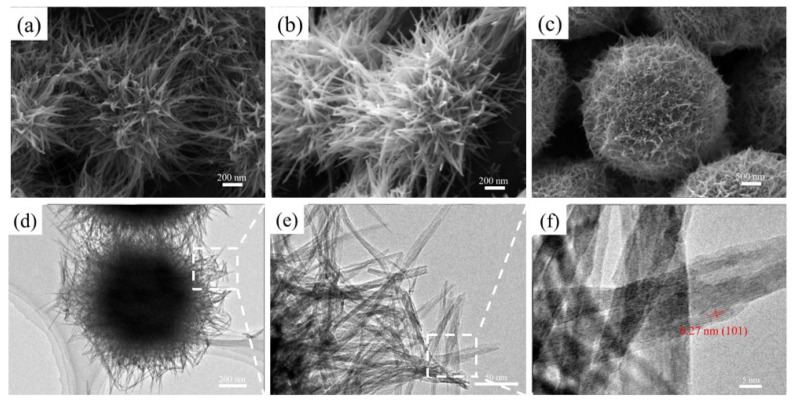
SEM images of, (**a**) RTMOA1, (**b**) RTMOA3, and (**c**) RTMOA5, and (**d**–**f**) TEM images of RTMOA3.

The SEM and TEM morphologies of RTMOA prepared at room temperature are shown in Figure 2. It was found that the microstructure of Ag-doped MnO_2_ prepared at room temperature is an urchin-like structure formed by overlapping extremely fine nanowires. The diameter of the sphere of samples is about 2 μm. The interplanar crystal spacing of the nanowires is about 0.27 nm, which is consistent with the (101) crystal face of α-MnO_2_ in the JCPDS 44-0141 data.

**Figure 3:**
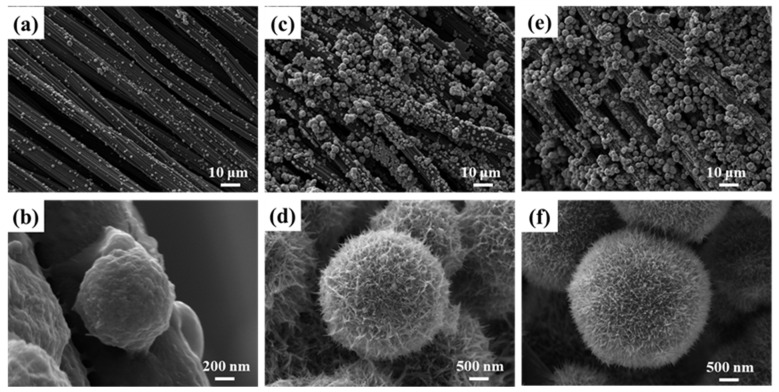
SEM images of (**a**,**b**) CRTMOA1, (**c**,**d**) CRTMOA3, and (**e**,**f**) CRTMOA5.

Figure 3 shows the surface morphology of MnO_2_ grown on the surface of CC under different Ag^+^ doping concentrations. As shown in Figure 3a,b, when the concentration of Ag^+^ is very low, a very small number of MnO_2_ spheres grow on the carbon fiber surface of the CC, the diameter of the spheres is about 1 μm, and most of the carbon fiber is bare. After increasing the Ag^+^ concentration, it can be seen from Figure 3c,d that urchin-like MnO_2_ was uniformly distributed on the surface of CRTMOA3, and the diameter of the spheres increased significantly, to about 2–3 μm. After the Ag^+^ concentration was further increased, it can be observed from Figure 3e,f that the diameter of the urchin-like MnO_2_ spheres on the surface of CRTMOA5 were still about 3 μm, and the surface was covered by nanowires that are denser than CRTMOA3. This is because when the concentration of Ag^+^ is too low, the diameter of the generated MnO_2_ nanowires is relatively large, and the length is slightly short. After increasing the proportion of AgNO_3_, the increasing concentration of Ag^+^ promoted polar growth of the nanowires, resulting in the formation of larger and more compact urchin-like structures. The formation mechanism of this urchin-like MnO_2_ structure can be divided into the following two-step growth process [38]: (1) In the initial phase, with high concentrations of reactants and Ag^+^, Ag^+^ induces homogeneous catalysis to form MnO_2_ spheres formed by self-assembly of MnO_2_ nanowires. (2) As the reaction progresses, the concentration is low due to the capture of reactants and effective Ag^+^, resulting in the heteroepitaxial growth of MnO_2_ nanowires on the surface of MnO_2_ spheres. The diameters of Ag@MnO_2_ particles loaded on the surface of the CC are slightly smaller than those of pure Ag@MnO_2_. One of the reasons is that the presence of CC layers provides a large 2D platform to disperse Ag^+^ active sites so that more active sites can produce more crystal nuclei. For the same reactant concentration, the number of Ag@MnO_2_ spheres is higher, and the corresponding volume is smaller. Another reason is that the carbon fibers in the CC isolate the crystal nuclei from each other and prevent the MnO_2_ crystal nuclei from bonding to form larger crystal nuclei.

**Figure 4:**
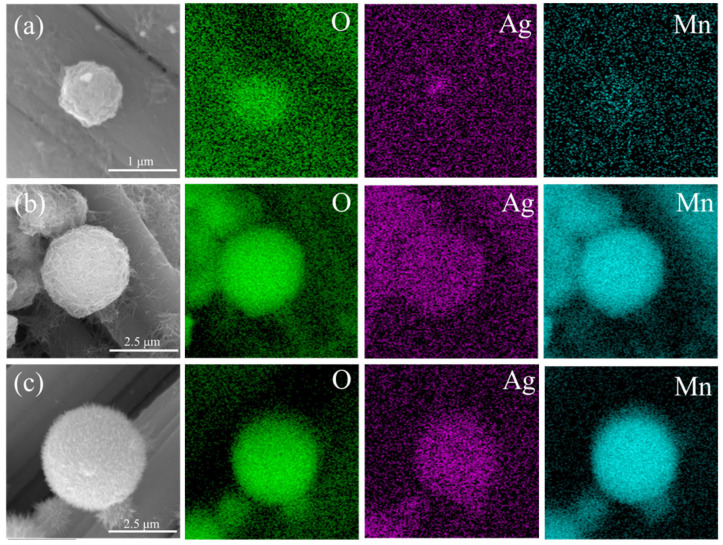
SEM images and EDS images of selected elements: O, Ag, and Mn of (**a**) CRTMOA1, (**b**) CRTMOA3, and (**c**) CRTMOA5.

Figure 4 shows the EDS mapping of CRTMOA1, CRTMOA3, and CRTMOA5. EDS images show the prepared Ag@MnO_2_ composed of Mn, O, and Ag. The enhanced Mn and O signals correspond to urchin-like MnO_2_ spheres. From Figure 4a, it can be seen that the urchin-like MnO_2_ spheres grown on the CC are relatively small, and it is difficult to observe the distribution of Ag, indicating that it is difficult to load MnO_2_ spheres onto the surface of the CC under this condition, and Ag is also difficult to load on the surface of MnO_2_. The EDS images of CRTMOA3 show significant differences compared with CRTMOA1, with clearer images of Mn and O elements, indicating the growth of more MnO_2_ spheres with larger diameters and uniform element distribution on the CC. Meanwhile, the image of Ag proves that compared with CRTMOA1, the surface of CRTMOA3 successfully loaded more and more uniform Ag atoms. In the images of CRTMOA5, the density of element distribution is higher than that of CRTMOA3, confirming the more compact urchin-like structures displayed in SEM images. However, the distribution of Ag did not significantly increase, indicating that the proportion of Ag in CRTMOA5 began to be excessive, and some Ag did not successfully load onto the surface of MnO_2_.

### 3.2. Chemical Structure Analysis

**Figure 5:**
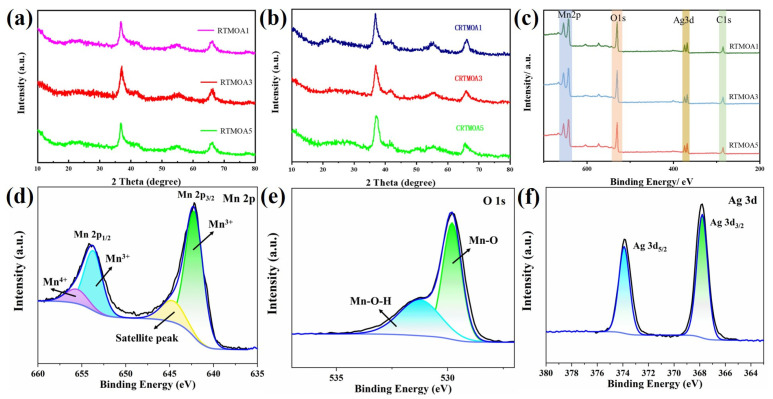
XRD spectra for (**a**) RTMOA1, RTMOA3, and RTMOA5, and (**b**) CRTMOA1, CRTMOA3, and CRTMOA5. (**c**) XPS full spectra for RTMOA1, RTMOA3, and RTMOA5. High-resolution (**d**) Mn 2p, (**e**) O 1s, and (**f**) Ag 3d XPS spectra for RTMOA3.

The XRD spectra of RTMOA prepared at room temperature are shown in Figure 5a. It can be observed that three samples exhibit significant diffraction peaks at 37.52°, 41.97°, 56.37°, and 66.69°, corresponding to the (211), (301), (600), and (112) crystal planes of α-MnO_2_ (JCPDS 44-0141). No other types of MnO_2_ peaks were observed, indicating that the prepared samples are only composed of α-MnO_2_. After silver doping, some diffraction peaks on crystal planes disappear because the addition of silver promotes the polar growth of nanowires, resulting in enhancement of the main peak corresponding to the plane and weakening of the diffraction peaks on other crystal planes. Figure 5b shows the XRD spectra of CRTMOA1, CRTMOA3, and CRTMOA5. The major diffraction peaks of MnO_2_ (JCPDS 44-0141) were found in all three composite spectra, which proved that highly crystalline MnO_2_ was successfully grown on CC. MnO_2_ produced by adding CC showed similar characteristics to MnO_2_ prepared at room temperature, indicating that the growth of MnO_2_ was not affected by adding CC. Some of the MnO_2_ diffraction peaks with low intensity disappear because they grow on the carbon cloth matrix, and the wide peaks of the CC cover up the weak peaks of the MnO_2_. With the increase of Ag-doping concentration in MnO_2_, the diffraction peak intensity of MnO_2_ decreases, indicating that the crystallinity decreases. No new peaks appear in the XRD spectra after doping, which indicates that Ag is evenly distributed in MnO_2_, and some Ag can be incorporated into the lattice of α-MnO_2_. In addition, it is found that the XRD peaks after doping have a slight shift to a lower angle, which indicates that the lattice expansion is caused by the doping into the lamellar spacing. These diffraction peak changes reveal that Ag is effectively doped into MnO_2_ nanowires. Figure 5c shows the XPS full spectra of RTMOA1, RTMOA3, and RTMOA5. According to the full spectra of XPS, three main peaks (C1s as calibration peak) appeared at binding energies of 378–364, 532, and 660–635 eV, corresponding to Ag 3d, O 1s, and Mn 2p, respectively, indicating that Ag@MnO_2_ was successfully prepared. Figure 5d–f shows high-resolution Mn 2p, O 1s, and Ag 3d XPS spectra for RTMOA3.

**Figure 6:**
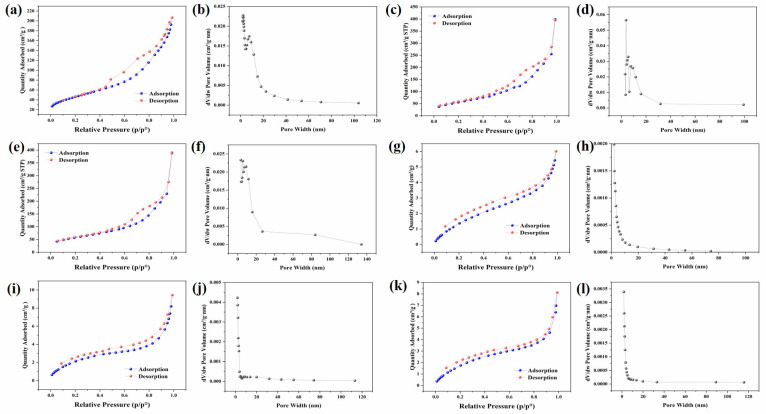
BET analysis of (**a**) RTMOA1, (**c**) RTMOA3, (**e**) RTMOA5, (**g**) CRTMOA1, (**i**) CRTMOA3, and (**k**) CRTMOA5, and pore-size distributions of (**b**) RTMOA1, (**d**) RTMOA3, (**f**) RTMOA5, (**h**) CRTMOA1, (**j**) CRTMOA3, and (**l**) CRTMOA5.

Figure 6 shows the nitrogen absorption–desorption isotherms with pore-size distributions of RTMOA and CRTMOA. The adsorption–desorption isotherms of RTMOA all show type IV isotherms, which proves that they are all mesoporous materials and the adsorption and desorption process is almost completely reversible. The pore sizes of RTMOA are concentrated within about 10 nm, and all of them have high specific surface areas of 169.008, 160.568, and 158.192 m^2^/g, respectively. Owing to the increase in Ag^+^ concentration, the catalytic effect becomes better, resulting in an increase in the density of the material and a decrease in the specific surface area of the material. Figure 6g–l shows the nitrogen absorption–desorption isotherms with the pore-size distributions of CRTMOA1, CRTMOA3, and CRTMOA5. The isotherms of three materials are type II, and the pore size is concentrated at about 2 nm. The pore sizes of CRTMOA become smaller relative to RTMOA because the proportion of CC in the composite material is relatively large, and the pore size of CC is concentrated at about 2 nm. The specific surface areas of the three materials were 6.906, 8.792, and 8.222 m^2^/g, respectively, showing a trend of first rising and then decreasing. This is because the catalytic effect is not obvious when the concentration of Ag^+^ is low, and very few MnO_2_ spheres grow on the CC in CRTMOA1, so the specific surface area of the composite is closer to the specific surface area of the CC itself. However, uniform MnO_2_ spheres were successfully grown on the CC in both CRTMOA3 and CRTMOA5, so the specific surface area of the two materials is relatively high. However, when the Ag^+^ concentration is high, MnO_2_ spheres grown on the CC are more dense, resulting in a slight decrease in CRTMOA5.

### 3.3. Electrochemical Characterization

**Figure 7:**
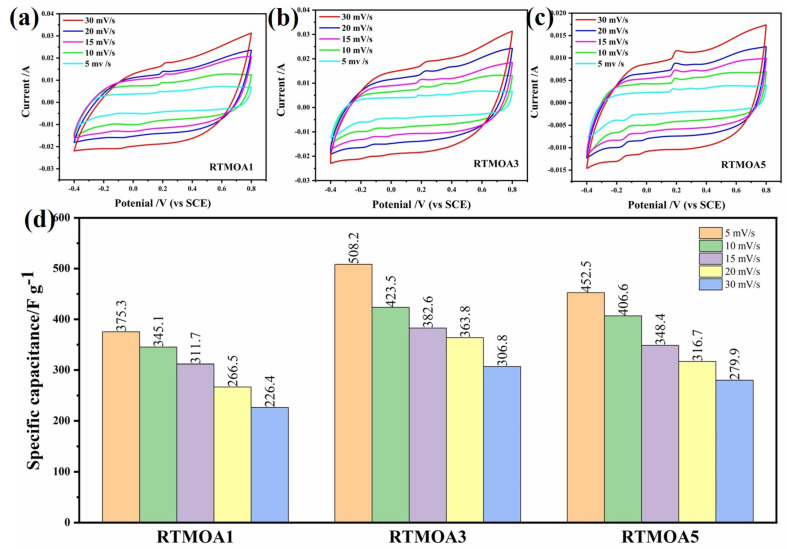
CV curves of (**a**) RTMOA1, (**b**) RTMOA3, and (**c**) RTMOA5, and (**d**) capacitance values of RTMOA1, RTMOA3, and RTMOA5 at different scan rates in 1M Na_2_SO_4_ solution.

Figure 7 shows the CV of Ag@MnO_2_ samples prepared at room temperature at different scan rates. The CV curve shapes of the three samples are basically consistent, and a pair of redox peaks appears at about 0.25 V and −0.15 V, indicating the pseudocapacitive characteristics of MnO_2_. The specific capacitance values of the three samples at different scan rates are shown in Figure 7d; the capacitance of RTMOA3 is higher than that of RTMOA1 and RTMOA5, and the capacitance values of RTMOA1, RTMOA3, and RTMOA5 are 375.3, 508.2, and 452.5 F/g at 5 mV/s, respectively. The capacitance values show a trend of first increasing and then decreasing, which is because the addition of Ag will increase the conductivity of the material, thus better displaying the pseudocapacitive characteristics of MnO_2_ and increasing the capacitance value of the material. However, Ag does not contribute to the capacitance of the composite, and if the amount of Ag in the composite is too high, the specific capacitance will be reduced owing to the increase in the total mass of the electrode.

**Figure 8:**
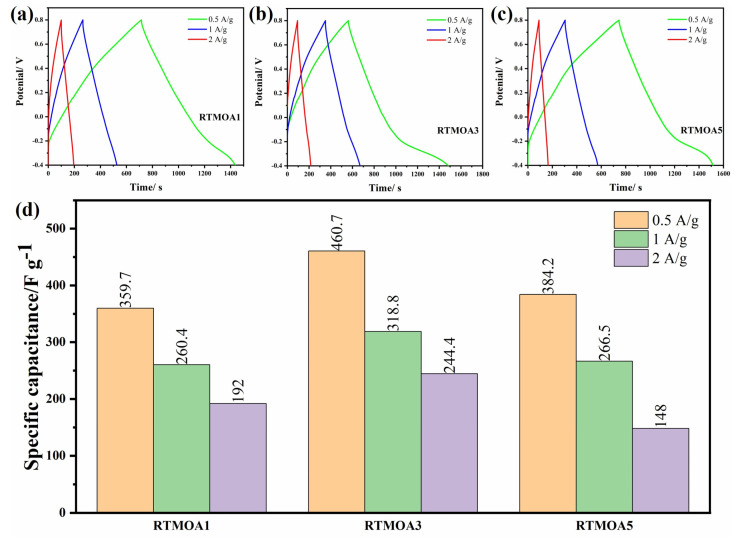
GCD curves of (**a**) RTMOA1, (**b**) RTMOA3, and (**c**) RTMOA5, and (**d**) capacitance values of RTMOA1, RTMOA3, and RTMOA5 at different current densities in 1M Na_2_SO_4_ solution.

Figure 8 shows the GCD curves of the above three samples, and RTMOA1 exhibits an approximate isosceles triangle shape at all three current densities. The GCD curve shapes of RTMOA3 and RTMOA5 are irregular triangles at 0.5 A/g, and the slopes of the curves begin to slow down when the voltage is about −0.15 V, which is because the material can give full play to the redox reaction at low current density, and the discharge time increases. The specific capacitance values of the three samples at different current densities are shown in Figure 8d, and the overall law is consistent with the specific capacitance calculated using the CV curve. The specific capacitance of RTMOA3 is the highest, reaching 460.7 F/g at 0.5 A/g.

**Figure 9:**
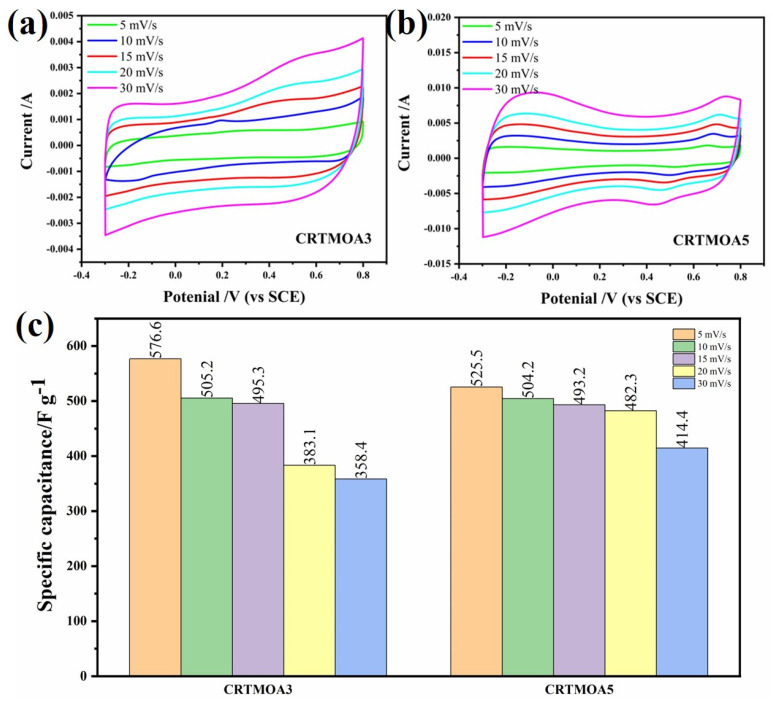
CV curves of (**a**) CRTMOA3 and (**b**) CRTMOA5, and (**c**) capacitance values of CRTMOA3 and CRTMOA5 at different scan rates in 1M Na_2_SO_4_ solution.

As shown in Figure 9, the CV curve for CRTMOA3 is approximately a parallelogram, indicating that the material has good reversible properties. Compared with CRTMOA3, CRTMOA5 shows a “depression” in the middle of the curve. This is because the density of MnO_2_ grown on the CC in the CRTMOA5 is too high, and the material cannot fully undergo redox reaction with the electrolyte. The pseudocapacitive characteristics of MnO_2_ are not fully displayed, resulting in a decrease in the capacitance value.

**Figure 10:**
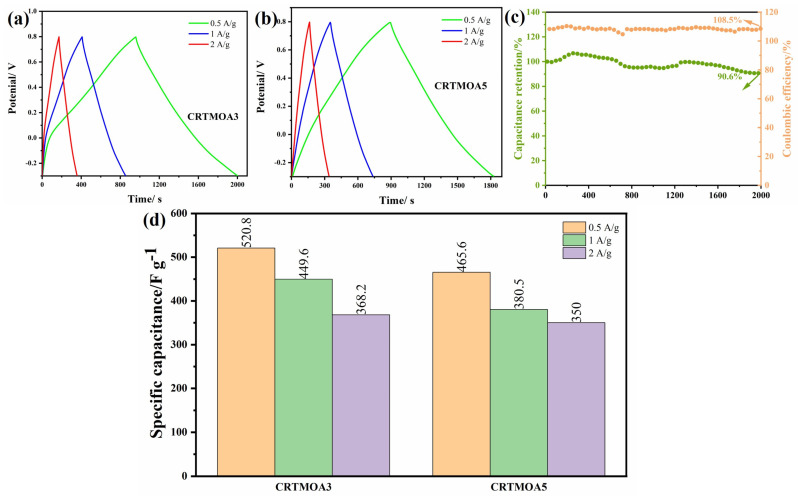
GCD curves of (**a**) CRTMOA3 and (**b**) CRTMOA5. (**c**) Changes in capacitance retention and Coulombic efficiency of CRTMOA3 after 2000 cycles at 1 A/g. (**d**) Capacitance values of CRTMOA3 and CRTMOA5 at different current densities in 1M Na_2_SO_4_ solution.

Figure 10 shows the GCD curves of CRTMOA3 and CRTMOA5 at different current densities. The GCD curves of the two samples exhibit approximate isosceles triangles at different current densities, indicating that MnO_2_ undergoes a very thorough redox reaction with the electrolyte, which is consistent with the characteristics exhibited by the CV curve. The capacitance of CRTMOA3 reached 520.8 F/g at 0.5 A/g, which further improved the specific capacitance compared with pure RTMOA3. This indicates that the self-supporting electrode prepared using in situ growth of MnO_2_ on CC is more conducive to fully exhibiting the pseudocapacitive properties of MnO_2_. In order to further characterize the electrochemical stability of the material, multiple charge and discharge tests were conducted on CRTMOA3 at 1 A/g. As shown in Figure 10c, the capacitance retention rate of CRTMOA3 after completing 2000 cycles is 90.6%, demonstrating good cyclic stability, which is attributed to the synergistic effect between carbon fibers and MnO_2_ spheres. MnO_2_ directly grows in situ on the surface of CC, resulting in a tighter binding between MnO_2_ and carbon fibers. During testing in aqueous electrolyte solutions, CC can prevent MnO_2_ from electrochemical dissolution and detachment. Moreover, CC can serve as a buffer to regulate the volume changes and related strain releases generated during the charge–discharge cycle process.

Table 1 shows the specific capacitance values of previously reported MnO_2_-based composite materials. By comparing these data with our work, it can be seen that this work used a simple one-step synthesis method to prepare MnO_2_-based flexible electrodes with high specific capacitance. 

## 4. Conclusions

Unlike previous hydrothermal synthesis of MnO_2_, this study synthesized Ag-doped MnO_2_ nanowires on carbon cloth at room temperature. The material has an urchin-like structure and a high specific surface area, which is conducive to ion and charge transfer. The doping of Ag not only catalyzes the reaction but also improves the electrochemical performance of the material. In addition, directly synthesizing MnO_2_ on carbon cloth is beneficial for the tight bonding between MnO_2_ and carbon fibers, thereby reducing the detachment of MnO_2_ during the testing process. The specific capacitance of the material can reach 520.8 F/g at 0.5 A/g. After 2000 cycles at 1 A/g, the capacitance retention rate of CRTMOA3 is 90.6%, indicating good cyclic stability. These all indicate that this composite material has great potential in the application of flexible electrode materials for supercapacitors.

## Figures and Tables

**Table 1:** The comparison of specific capacitance between CRTMOA and other MnO_2_-based electrodes.

Sample	Electrolyte	Current Density (A/g)	C_s_ (F/g)	Ref
Ag_2_-MnO_2_	1M Na_2_SO_4_	1	350	[38]
MnO_2_-SHAC-3	1M Na_2_SO_4_	1	224.3	[39]
SGM-3	1M Na_2_SO_4_	0.2	260	[37]
MCE	1M Na_2_SO_4_	1	148	[40]
CRTMOA3	1M Na_2_SO_4_	0.5	520.8	This work

## Data Availability

Data are contained within the article.
